# Characterizing the Protein Isoforms of *foraging* (*for*), the PKGI Ortholog in *Drosophila melanogaster*

**DOI:** 10.3390/ijms241210219

**Published:** 2023-06-16

**Authors:** Oscar E. Vasquez, Aaron M. Allen, Anthony K.-C. So, Quynh H. Nguyen, Henry M. Krause, Joel D. Levine, Marla B. Sokolowski

**Affiliations:** 1 Department of Ecology and Evolutionary Biology, University of Toronto, Toronto, ON M5S 3B2, Canada; 2 Centre for Neural Circuits and Behaviour, Oxford University, Oxford OX1 3SR, UK; aaron.allen@dpag.ox.ac.uk; 3 Department of Biology, University of Toronto at Mississauga, Mississauga, ON L5L 1C6, Canada; anthony.kc.so@gmail.com (A.K.-C.S.); joel.levine@utoronto.ca (J.D.L.); 4 Department of Biochemistry, University of Toronto, Toronto, ON M5S 1A8, Canada; qhuong.nguyen@mail.utoronto.ca; 5 Department of Molecular Genetics, University of Toronto, Toronto, ON M5S 3E1, Canada; h.krause@utoronto.ca; 6 Child and Brain Development Program, Canadian Institute for Advanced Research (CIFAR), Toronto, ON M5G 1M1, Canada

**Keywords:** cGMP-dependent protein kinase, protein kinase G, PKG, *foraging* gene, protein isoforms, *Drosophila melanogaster*

## Abstract

The *foraging* (*for*) gene of *Drosophila melanogaster* encodes a cGMP-dependent protein kinase (PKG), which is a major effector of the cGMP signaling pathway involved in the regulation of behaviour and metabolic traits. Despite being well studied at the transcript level, little is known about the *for* gene at the protein level. Here, we provide a detailed characterization of the *for* gene protein (FOR) products and present new tools for their study, including five isoform-specific antibodies and a transgenic strain that carries an HA-labelled *for* allele (*for^BAC^::HA*). Our results showed that multiple FOR isoforms were expressed in the larval and adult stages of *D. melanogaster* and that the majority of whole-body FOR expression arises from three (P1, P1α, and P3) of eight putative protein isoforms. We found that FOR expression differed between the larval and adult stages and between the dissected larval organs we analyzed, which included the central nervous system (CNS), fat body, carcass, and intestine. Moreover, we showed that the FOR expression differed between two allelic variants of the *for* gene, namely, *for^s^* (sitter) and *for^R^* (rover), that are known to differ in many food-related traits. Together, our in vivo identification of FOR isoforms and the existence of temporal, spatial, and genetic differences in their expression lay the groundwork for determining their functional significance.

## 1. Introduction

cGMP-dependent protein kinase (PKG) is a major effector of the cGMP-signaling pathway, whose structure and function are conserved in animals. PKG has multiple functions (pleiotropic) and mediates the regulation of numerous physiological processes. In mammals, physiological processes associated with PKG function include vascular smooth muscle relaxation, platelet aggregation, and neuronal plasticity [1,2,3,4]. PKG has also been studied extensively in the vinegar fly (*Drosophila melanogaster*), where it exhibits pleiotropy and affects multiple phenotypes involved in behaviour, neurotransmission, and renal fluid transport [5,6,7]. How PKG regulates its various phenotypes is not entirely understood; however, the use of multiple PKG proteins that are encoded by different genes (isozymes) or by the same gene through alternative splicing events (isoforms) plays a major role [3].

In mammals, the PKG function is performed by three PKG proteins (PKGIα, PKGIβ, and PKGII). PKGIα and PKGIβ are isoforms encoded by the same gene, namely, *prkg1*, whereas PKGII is encoded by a different gene, namely, *prkg2* [3]. Structurally, mammalian PKGs differ the most in their N-terminal domains, and studies of PKGIα and PKGIβ especially show that the N-terminal domain of PKG underlies many of the differences observed in PKG function [8,9,10,11,12]. PKGs canonically possess three domains: (i) an N-terminal regulatory domain, (ii) a cGMP-binding regulatory domain that contains two tandem cGMP-binding domains (CNB-A and -B), and (iii) a catalytic domain responsible for the phosphorylation of substrates. Within the N-terminal regulatory domain are a conserved leucine/isoleucine zipper (ILz) motif and an autoinhibitory (Ai) sequence. In mammals, alternative splicing of *prkg1* transcripts results in the translation of two protein isoforms, namely, PKGIα and PKGIβ, that differ only in their N-terminal domains, which are 89 and 104 amino acids in length, respectively [13,14]. Despite varying only in their first ~100 amino acids and having identical sequences for their two CNB and catalytic domains, PKGIα and PKGIβ differ in their cGMP binding affinity, substrate specificity, activation state, and subcellular localization [3]. PKGIα and PKGIβ also differ in the phenotypes they regulate [15].

The expression of multiple PKG proteins in distinct tissues and at different times is also observed in *D. melanogaster* [3,16,17,18,19,20]. Three PKG genes are present in the *D. melanogaster* genome and, of these, the *foraging* (*for*) gene encodes multiple putative protein isoforms [5,21,22,23]. The *for* gene is orthologous to mammalian *prkg1* and has long served as a model for studying the regulation of behaviour and its evolution [21,22,24,25]. The link between *for* and behaviour was first demonstrated in 1989 [5] when differences in larval foraging behaviour identified in 1980 [26] were shown to be largely influenced by allelic variation at the *for* locus. Specifically, it was shown that individual larvae that carry a sitter *for* allele (*for^s^*) travel shorter distances on their food, namely, a yeast paste, compared with individuals that carry a rover *for* allele (*for^R^*). Sitter and rover larvae also differ in many other *for*-regulated traits that include food intake, fat levels, and thermal nociception [18,27]. Overall, a combination of studies on sitter, rover, and *for* genetic mutants highlighted *for*’s pleiotropic nature [5,6,18,25,27,28]. How *for* regulates its pleiotropy is not fully understood; however, the availability of many *for* mutants and molecular tools [25] and the genetic tractability of *D. melanogaster* [29,30,31] make *for* an attractive model to study PKG function.

Recently, it was shown that *for*’s pleiotropy can be explained in part by the tissue-specific expression of its transcripts [16,17]. The *for* gene is structurally complex and contains four minimal promoters associated with four transcription start sites (TSSs) that regulate the spatial and temporal expression of 22 alternatively spliced transcripts [17,20,22,27]. The *for* gene transcripts differ only at their 5′ ends and encode multiple putative protein products (FOR) that vary in their N-terminal sequence [27]. Although much is now known about the transcriptional regulation of *for*, little is known about *for* at the protein level. Previous studies of *Drosophila* homogenates detected multiple FOR-specific bands via Western blotting [21,27,32,33,34]. However, a detailed in vivo characterization of the isoform identity of previously detected FOR-specific bands and their spatial and temporal expression is lacking. Such kinds of investigations are needed to gain a better and more complete understanding of how *for* regulates phenotypes and achieves its pleiotropy.

Here, we present a detailed characterization of the *for* gene at the protein level. We proceeded by exploring the amino acid sequences of the potential FOR isoforms encoded by the many *for* transcripts. We established which FOR isoforms were expressed in vivo and when and where they were expressed. Moreover, we characterized FOR expression in sitter and rover *for* allelic variants and identify a difference in FOR isoform expression that could underlie *for*-associated differences in phenotypes observed in these two strains [25]. Overall, our results deepen our understanding of *for* expression at the molecular level, shed light on the protein isoforms involved in regulating *for* function, and lay the foundation for future studies of isoform-specific regulation of *for* phenotypes. Our findings also contribute to our understanding of PKG structure in other species.

## 2. Results

### 2.1. The foraging Gene Encoded Eight Putative Protein Isoforms that Differed at Their N-Termini

**Figure 1:**
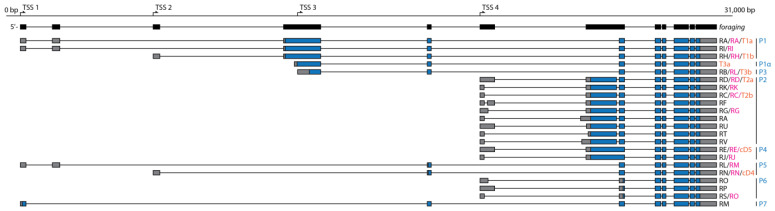
Summary of the 22 annotated *for* transcripts and the longest ORFs they encode in *for^s^* considering only AUG start codons. Exons and the four transcription start sites (TSSs) of the *for* gene are illustrated in black. Transcripts are colour-coded to show the coding sequence in blue and untranslated regions in grey. Transcript names from three sources are provided: black from [27], fuchsia from FlyBase, and orange from [22]. The names of the putative protein isoforms encoded by each transcript are in blue. In total, *for* transcripts encoded eight distinct ORFs, although the P7 ORF was specific to the *for^s^* allele.

We started our investigation by performing an in silico analysis of annotated *for* gene transcripts to identify and characterize all theoretical protein isoforms. We analyzed the sitter sequence first and examined a total of 22 unique *for^s^* allele transcripts [22,27,35]. Considering only AUG start sites and assuming that translation began at the first methionine codon, we found eight distinct open reading frames (ORFs) whose translation yielded eight putative FOR isoforms (Figure 1). We labelled these putative isoforms P1, P1α, P2, P3, P4, P5, P6, and P7, for protein (P) one through seven since most *for* ORFs were encoded by more than one transcript. Isoforms P1α and P7 are not currently annotated in FlyBase [36], which is a *D. melanogaster* database of genes and genomes. Interestingly, P1α was previously predicted to be translated from a *for* transcript with minor expression [22]. Isoform P7 is suggested to be a *for^s^*-specific theoretical isoform and is discussed later.

**Figure 2:**
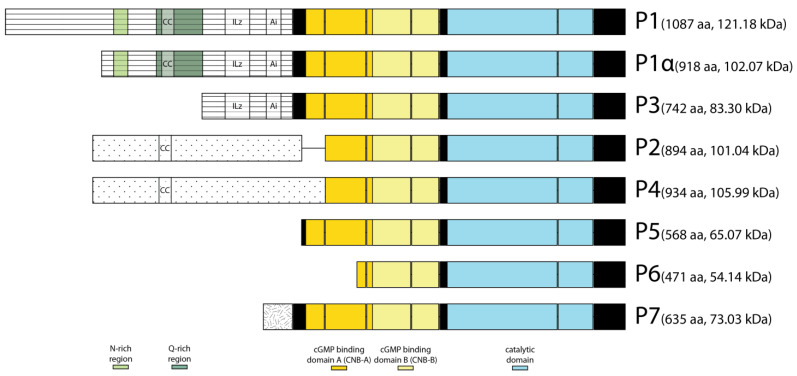
Putative FOR isoforms differed at their N-termini. Schematic of the eight putative FOR isoforms encoded by *for^s^* transcripts and their predicted domains and motifs. Identical amino acid sequences are illustrated with the same fill pattern and aligned. The leucine/isoleucine zipper (ILz) motif and autoinhibitory (Ai) sequence were predicted from a sequence alignment with PKGI [37]. Coiled-coil (CC) motifs and N- and Q-rich regions were predicted using PHMMER [38].

Amino acid sequence alignment of the eight putative FOR isoforms with mouse PKGI showed that FOR isoforms differed at their N-terminal domains and shared identical sequences for their two CNBs and catalytic domains (Figure 2). The extent to which FOR isoforms differed in their N-terminal domain sequence varies. Since each putative FOR isoform contained a complete catalytic domain, each had the capacity to phosphorylate downstream targets. Most putative FOR isoforms could be categorized into two groups based on the shared amino acid sequence of their N-terminal domains. One group included P1, P1α, and P3. The N-terminal domains of these three isoforms were encoded by exon 4 and differed only in their sequence length. The coding sequences for P1α and P3 were nested within that of P1, and their translation start codons occurred at M170 and M346 of the *for^s^* P1 ORF, respectively. The N-terminal domains of P1, P1α, and P3 contained putative sequences for an ILz motif and Ai sequence and both motifs showed high sequence similarity to that of mammalian PKGI [37]. We used PHMMER and ProSite to identify other putative motifs within the N-terminal domain of P1 and found most of this sequence to be intrinsically disordered [38]. PHMMER identified a coiled-coil motif between amino acids 277 and 297, while ProSite identified an asparagine-rich region between residues 191 and 216 and a glutamine-rich region between residues 267 and 346. ProSite also identified various sites for phosphorylation by PKC, CK2, and PKA/PKG, and for N-linked glycosylation (Table S1).

The second group of putative FOR isoforms included P2 and P4. The N-terminal domains of these two isoforms were encoded by exon 7, but a difference in splicing added 40 amino acids to the N-terminal domain of P4 [27]. The N-terminal domain sequences of P2 and P4 lacked a conserved ILz motif and Ai sequence and were mostly intrinsically disordered (Figure 2). Analysis of the P4 N-terminal domain with PHMMER identified a coiled-coil motif between residues 118 and 138, while ProSite identified various putative sites for phosphorylation by PKC, CK2, and PKA/PKG, and for N-linked glycosylation (Table S2).

### 2.2. Many FOR Polypeptides Were Observed In Vivo and Their Expressions Differed between Developmental Stages

**Figure 3:**
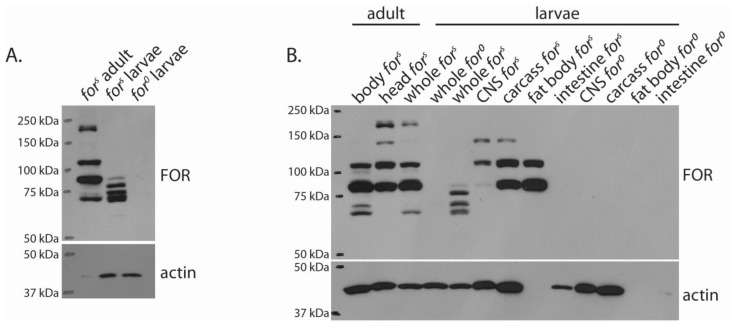
FOR expression differed between developmental stages and organs within an individual. Western blot of the FOR expression in whole-body homogenates from *for^s^* adult and mid-third instar larvae (**A**). Mid-third instar *for* null (*for^0^*) larvae were used as negative controls. Actin was used as a loading control. Western blot of the FOR expression in adult bodies and heads, as well as in dissected larval organs from *for^s^* individuals (**B**). *for^0^* larval organs were used as negative controls. Blots probed with anti-FOR(3).

We began our in vivo characterization of FOR by examining the whole-body expression in *for^s^* larvae and adults. Blots probed with anti-FOR(3), which is an antibody specific and common to all predicted FOR isoforms [34], revealed that many FOR bands were present in *for^s^*, but not in *for* null (*for^0^*), individuals (Figure 3A). Only one polypeptide, running at 150 kDa, possessed a molecular weight greater than predicted by the eight FOR isoform sequences (Figure 2). We also found that the expression of FOR isoforms differed greatly between the larval and adult developmental stages. This was most obvious for the expression of the higher molecular weight FOR polypeptides that were detected in the whole-adult, but not larval, lysates. The adult and larval FOR expression we observed also differed from that reported in embryos [33]. Generally, we observed that the expression of FOR did not differ between the second and third instar larvae (aged 72 h after egg hatch (AEH)) (Figure S1). FOR expression changed by 90 h AEH and continued to change as larvae enter the non-feeding wandering stage at 100 h AEH. The FOR expression changed again in the adult stage. Overall, our results indicate that FOR isoforms and expression levels differed between developmental stages.

### 2.3. The Expression of FOR Differed between Tissues

Transcript expression data strongly suggest that FOR isoforms are expressed in an organ-specific manner [16,17,20,39]. We investigated FOR isoform-specific expression with a series of Western blots of dissected adult and larval preparations. In contrast to larval dissections, which were precise and segregated the CNS, carcass (epithelia and body wall muscle), fat body, and intestines, adult dissections were crude and entailed separating heads from bodies. Results from Western blots probed with anti-FOR(3) showed that FOR expression differed between adult heads and bodies and larval organs (Figure 3B). In adults, the 150 kDa FOR polypeptide was enriched in heads but not bodies, whereas the 74 kDa and 71 kDa FOR polypeptides were enriched in bodies but not heads. In larvae, FOR expression differed between all organs that we analyzed. FOR was detected in the CNS, carcass, and fat body of fed larvae but not in their intestine. The detection of certain FOR polypeptides, such as the 108 kDa polypeptide, in dissected larval organs but not in the whole body also highlighted the difference in expression between *for* proteins. Overall, these results showed that FOR expression differed between tissues in adults and larvae.

### 2.4. The Majority of Whole-Body FOR Expression Was P1, P1α, and P3

**Figure 4:**
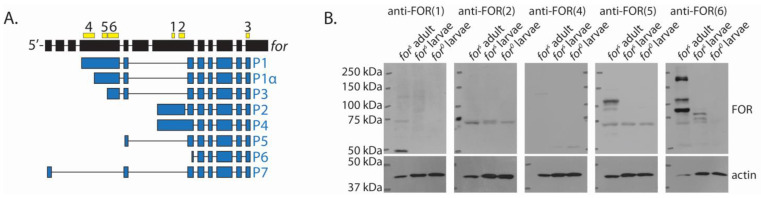
The majority of FOR expression in adult and mid-third instar larvae was P1, P1α, and P3. Schematic of the six antigenic regions used to generate each FOR antibody, anti-FOR(1) to anti-FOR(6) (**A**). Antigenic regions are in yellow. Exons for the *for* gene are in black. FOR isoform coding sequence is in blue. FOR isoforms are labelled P1 to P7. FOR isoforms have the following theoretical molecular weights: 121 kDa for P1, 102 kDa for P1α, 101 kDa for P2, 83 kDa for P3, 106 kDa for P4, 65 kDa for P5, 54 kDa for P6, and 73 kDa for P7. Western blots of whole-body homogenates from *for^s^* adults and mid-third instar larvae probed with antibodies expected to detect specific FOR isoforms (**B**). Mid-third instar *for^0^* larvae were used as a negative controls. Actin was used as a loading control. The antibody used to probe each blot is labelled above.

Although most of the FOR polypeptides detected in whole-adult and larval lysates had molecular weights that were close to what was expected, we confirmed their isoform identity using isoform-specific FOR antibodies. We used five new antibodies to immunodetect FOR in adult and larval lysates. Each new antibody was designed to detect a distinct N-terminal antigenic region in P1, P1α, P2, P3, or P4 (Table 1). Briefly, anti-FOR(1) and anti-FOR(2) recognized P2 and P4, anti-FOR(4) recognized P1, anti-FOR(5) recognized P1 and P1α, and anti-FOR(6) recognized P1, P1α, and P3 (Figure 4A). For each FOR antibody, we considered bands to be FOR-specific only if they were detected in wild-type adult and larval lysates and not in *for^0^* larval lysates.

Western blots immunodetected with our isoform-specific antibodies showed that the majority of adult and larval FOR expression was P1, P1α, and P3 (Figure 4B). Anti-FOR(1) and anti-FOR(2) did not detect any FOR-specific bands in whole-adult or larval lysates, whereas anti-FOR(6) detected the same FOR-specific bands as anti-FOR(3) (Figure 2). We used anti-FOR(5) and anti-FOR(4) to confirm the presence of P1 and P1α. anti-FOR(5) detected FOR bands at 128 kDa, 108 kDa, and 103 kDa in adults, and at 108 kDa in larvae. anti-FOR(4) detected the FOR band at 128 kDa in adults. Together, these results suggest the 128 kDa FOR band is a full-length P1 isoform and the FOR bands at 108 kDa and 103 kDa are P1α. Isoform P1 had a theoretical molecular weight of 121 kDa, while P1α had a theoretical weight of 102 kDa.

Our results from the Western blots probed with anti-FOR(6) also suggest that truncated variants of P1, P1α, or P3 were generated in vivo and that P3 was modified to run at 150 kDa. We did not test whether these truncated isoforms were generated via translational regulation of *for* transcripts or post-translational protein cleavage of P1, P1α, or P3. The lack of detectable bands using anti-FOR(1) and anti-FOR(2) suggests these antibodies did not efficiently bind in vivo P2 and P4 isoforms. Alternatively, P2 and P4 isoforms were not translated during the adult or mid-third instar larval stages, or their expression was too low to be detected in the whole-body homogenates. The absence of bands associated with isoforms P5 and P6 in the whole-body homogenates further suggests that these isoforms were not translated while larvae were in the fed state, or that their expression was either too low or limited to a specific subset of regions to be detected.

### 2.5. LC-MS/MS of FOR Bands Corroborated Their Isoform Identity

**Figure 5:**
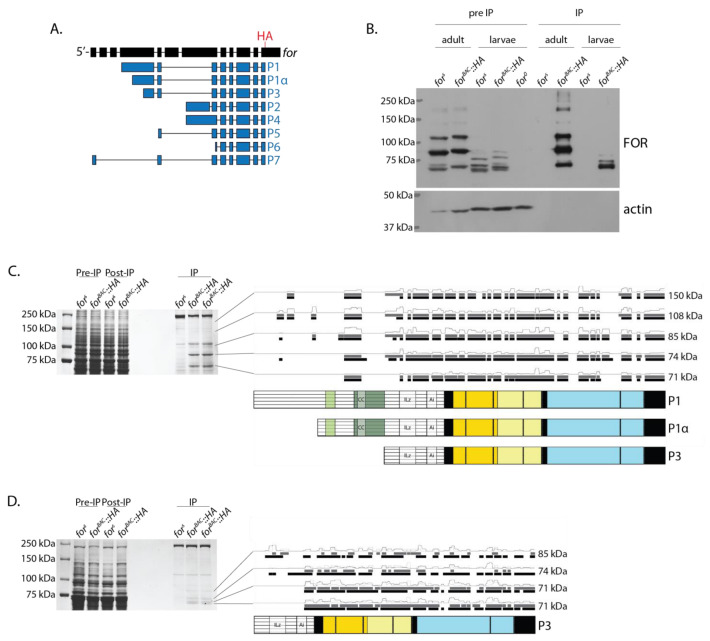
Purified FOR bands from adult and mid-third instar larvae were P1, P1α, and P3. Results of the adult and larval FOR::HA bands analyzed using LC-MS/MS corroborated the data from Western blots of whole-body lysates immunodetected with isoform-specific antibodies. Schematic of the *for^BAC^::HA* transgene used for purifying FOR (**A**). The sequence for an HA epitope tag (red) was inserted at the C-terminal end of the *for* coding sequence. Western blot of immunoprecipitated FOR::HA protein from fed adults and mid-third instar *for^BAC^::HA* larvae (**B**). *for^s^* larvae were used as negative controls for anti-HA immunoprecipitation and *for^0^* larvae were used as negative controls for the Western blotting. The top shows the FOR expression as detected using anti-FOR(3). The bottom shows actin as a control. SDS-PAGE gel showing the adult FOR::HA bands that were analyzed using LC-MS/MS and a summary of their detected peptide fragments (**C**). SDS-PAGE gel showing the larval FOR::HA bands that were analyzed using LC-MS/MS and a summary of their peptide fragments (**D**). In both adults and larvae, bands were analyzed in duplicate and digested with trypsin (black bars) or chymotrypsin (grey bars). Amino acid relative abundances are provided above the detected peptide fragments for each band. For all FOR isoform schematics: N-rich regions are illustrated in light green, Q-rich regions in dark green, CNB domains in yellow, and catalytic domains in blue.

We performed liquid chromatography–tandem mass spectrometry (LC-MS/MS) on adult and larval FOR bands excised from SDS-PAGE as a complementary approach to confirm their isoform identity. Since our anti-FOR(3) antibody did not efficiently pull down FOR, we generated a transgenic line that expressed FOR tagged with HA at its C-terminus (*for^0^*; *{for^BAC^::HA}*) and examined its FOR polypeptides (Figure 5A). The *for^BAC^::HA* allele rescued the late pupal lethality of homozygous *for^0^* individuals and recapitulated FOR expression in adults and larvae, just as other previously generated *for^BAC^* alleles do (Figure 5B) [27,32].

Since the *for* allele used in the *for^BAC^::HA* construct was that of the FlyBase reference genome line (RGL, rel. FB2020_06.37) [35], we performed an in silico analysis of RGL sequence and found several amino acid sequence differences between the *for^BAC^::HA* and *for^s^* alleles (discussed later) that we considered in the MS analysis of the *for^BAC^::HA* protein bands, hereafter referred to as FOR::HA. In total, we purified nine FOR::HA bands from adult and larval lysates: five bands from adults and four bands from larvae (Figure 5C,D). None of the purified FOR::HA bands were present in the *for^s^* control lysates and all bands were immunodetected with anti-FOR(3) on Western blots (Figure 5B). The LC-MS/MS results showed that every band analyzed was enriched for FOR::HA. In agreement with our Western blot data of FOR expression, only P1, P1α, and P3 N-terminal peptides were identified. To estimate the likelihood that a given amino acid sequence was present in a FOR::HA band, we calculated the amino acid relative abundances, which ranged from 0.0 to 1.0 within each sample (Figure 5C,D). In adults, peptides specific to the P1 N-terminal domain were detected in every band analyzed (Figure 5C). However, the relative abundances of certain peptide fragments specific to the P1 N-terminal domain differed substantially between the bands hypothesized to have their sequence given our results from Western blots using isoform-specific antibodies. This suggests that the detection of some P1 N-terminal fragments in the LC-MS/MS experiments might have resulted from contamination from previous runs with higher-molecular-weight samples. In larvae, P3 N-terminal domain fragments were detected in every band analyzed (Figure 5D).

A comparison of N-terminal amino acid relative abundances between samples in adults suggests that the 128 kDa band was P1::HA, the 108 kDa band was P1α::HA, the 85 kDa band was P3::HA, and that the 71 kDa band was a truncated P1::HA, P1α::HA, or P3::HA isoform with an amino acid sequence that likely began at the C-terminal of GSAAGCAGTG (P1 amino acids 434-443) (Figure 5C). The results were not as clear for deducing whether the 150 kDa band was P1::HA, P1α::HA, or P3::HA. A comparison of N-terminal amino acid relative abundances between samples in larvae suggests that the 83 kDa band was P3::HA, while the 74 kDa, 71 kDa, and 69 kDa bands were truncated P1::HA, P1α::HA, or P3::HA isoforms whose amino acid sequences likely started near ALGISAEPQS (P1 amino acids 477–486) (Figure 5D). A theoretical isoform with an amino acid sequence starting at ALGISAEPQS would be 611 amino acids long and have a molecular weight of 69.89 kDa.

### 2.6. LC-MS/MS Revealed Conserved Phosphorylation of the Active Loop Residues in FOR

**Figure 6:**
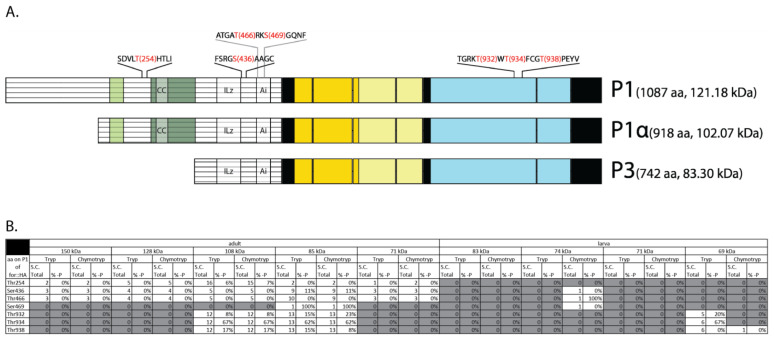
Phosphorylation sites in P1, P1α, and P3 detected using LC-MS/MS. Schematic illustrating the location of each phosphorylation site (red) detected using LC-MS/MS (**A**). Table summary of each phosphorylation site, including the number of times each site was detected (spectral count, S.C., total) per sample and the extent to which each site was phosphorylated as a percentage (%-P) (**B**). For all FOR isoform schematics: N-rich regions are illustrated in light green, Q-rich regions in dark green, CNB domains in yellow, and catalytic domains in blue.

LC-MS/MS of adult and larval FOR::HA bands also revealed phosphorylation sites in P1, P1α, and P3. Overall, seven phosphorylation sites were detected. These occurred at T254, S436, T466, S469, T932, T934, and T938 in P1 (Figure 6A). Amino acid T254 occurred within the N-terminal domain of P1 and P1α, and S436, T466, and S469 occurred within the N-terminal domain of P1, P1α, and P3. Specifically, S436 was found near the ILz motif, whereas T466 and T469 were present in the Ai sequence. Residues T932, T934, and T938 were found in the activation loop of FOR’s kinase domain [37]. Of all the phosphorylation sites detected using LC-MS/MS, only T466 was predicted in silico by ProSite (as a PKC phosphorylation site). FOR’s N-terminal phosphorylation sites T254, S436, T466, and S469 are not conserved in mouse PKGIα or PKGIβ isoforms, whereas FOR’s kinase domain phosphorylation sites T932, T934, and T938 are conserved [40,41].

Interestingly, FOR’s in vivo phosphorylation sites differed in the extent to which each was phosphorylated (Figure 6B). P1, P1α, and P3 N-terminal sites T254, S436, T466, and S469 were phosphorylated at lower proportions and less consistently compared with kinase domain sites T932, T934, and T938. For example, the N-terminal site S436 was detected in every adult FOR::HA band analyzed using MS; however, phosphorylated-S436 (p-S436) was detected only in the 85 kDa band and at a proportion of 11% (1 of 9 counts). S436 had the highest proportion of phosphorylation out of all N-terminal phosphorylation sites that were detected more than once per sample. In comparison, kinase domain site T934 was detected and phosphorylated in every FOR::HA band analyzed. The proportion of detected p-T934 ranged between 62% and 67%. These results corroborate studies in mice that showed that N-terminal phosphorylation of PKGI is not detectable in vivo, whereas phosphorylation of T932, T934, and T938 orthologous sites is detectable in vivo [40,41]. The proportions of phosphorylated T932, T934, and T938 sites in FOR were similar across isoforms and between adults and larvae.

### 2.7. FOR Sequence Differed at Several Amino Acids in for^s^ and for^R^

**Figure 7:**
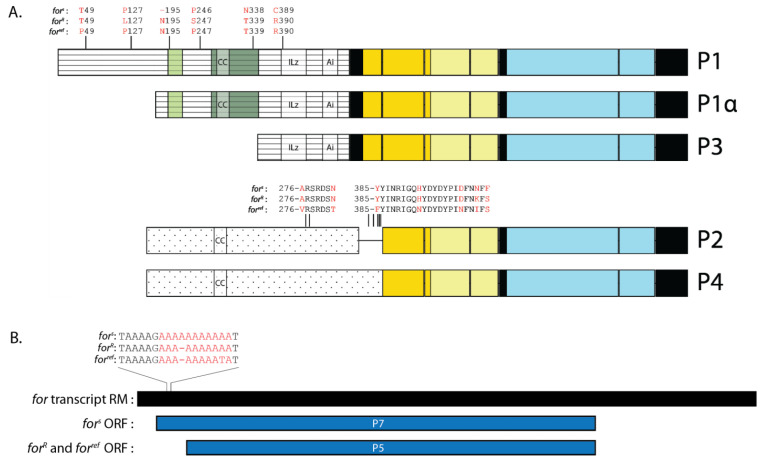
DNA sequence variation in *for^s^*, *for^R^*, and *for^ref^* led to allele-specific amino acid differences in P1, P1α, P3, P2, and P4, and a *for^s^*-specific P7 isoform. Schematic showing the location of each amino acid difference (red) present in *for^s^*, *for^R^*, and *for^ref^* (**A**). Differences in the amino acid sequence were not found in P5 or P6. Schematic of the DNA sequence variation (red) in *for^s^*, *for^R^*, and *for^ref^*, as well as its location within transcript RM (black), that leads to the translation of a *for^s^*-specific P7 isoform (dark blue) (**B**). Transcript RM in *for^R^* and *for^ref^* encoded isoform P5 (dark blue). For all FOR isoform schematics: N-rich regions are illustrated in light green, Q-rich regions in dark green, CNB domains in yellow, and catalytic domains in blue.

The *for^s^* and *for^R^* strains are well studied *for* allelic variants that differ in many of their *for*-regulated phenotypes. However, little is known about the differences between *for^s^* and *for^R^* at the protein level. Since the genomes of *for^s^* and *for^R^* have been sequenced, we compared the primary structure of their putative FOR isoforms to identify variations in the amino acid sequence [16]. In total, we found six amino acid substitutions between *for^s^* and *for^R^* protein isoforms and a single amino acid deletion in *for^s^* (Figure 7A). Four of the amino acid substitutions occurred within the N-terminal domain of P1 and two within the N-terminal domain of P4. The P1 amino acid substitutions occurred at positions 127, 247, 339, and 390. The amino acid positions 247 and 339 of P1 were shared with P1α, whereas position 390 was shared with P1α and P3. The P4 amino acid substitutions occurred at positions 404 and 406 and were not shared with P2. Of the six positions that differed between *for^s^* and *for^R^*, *for^BAC^::HA* shared the same amino acid identity as *for^s^* at three positions, and as *for^R^* at two positions. A single amino acid deletion was also found in P1 of *for^s^*, but not *for^R^* and *for^ref^*, and occurred at position 195. The isoforms from *for^s^* and *for^R^* also shared six amino acid substitutions that differed from *for^BAC^::HA* (Figure 7A). One of these amino acid substitutions occurred at position 49 in the N-terminal region of P1. The rest occurred at positions 276, 282, 385, 393, and 401 in the N-terminal region of P2 and P4. It is not known how these variations in FOR amino acid sequence affect protein function.

### 2.8. P7 Was a Putative FOR Isoform Specific to for^s^

Our analysis of FOR ORFs revealed that *for^s^* differed from *for^BAC^::HA* in that its RM transcript encoded a unique P7 isoform (Figure 7B). We analyzed transcript RM of *for^R^* and found it too encoded P5 and not P7. In *for^R^* and *for^BAC^::HA*, the largest ORF of transcript RM began at nucleotide position 288 and codes for P5. In *for^s^*, the insertion of an adenosine between nucleotide positions 138 and 148 extended the RM ORF at its 5′-end such that it began at nucleotide position 88. Translation of the RM ORF in *for^s^* yielded a protein with an extra 67 N-terminal amino acids, 53 of which generated a novel N-terminal domain. This unique isoform, namely, P7, was not encoded by any other *for* transcript and is suggested to be specific to *for^s^*.

According to the in silico investigation, the P7 amino acid sequence featured a complete CNB-A and an N-terminal domain that lacked a conserved ILz motif and Ai sequence. No probable motifs or sites for post-translational modifications (PTMs) were identified in the N-terminal domain of P7 using PHMMER and ProSite, respectively. It is not known how the N-terminal domain of P7 regulates its function.

### 2.9. FOR Expression Differed between for^s^ and for^R^ Larvae

**Figure 8:**
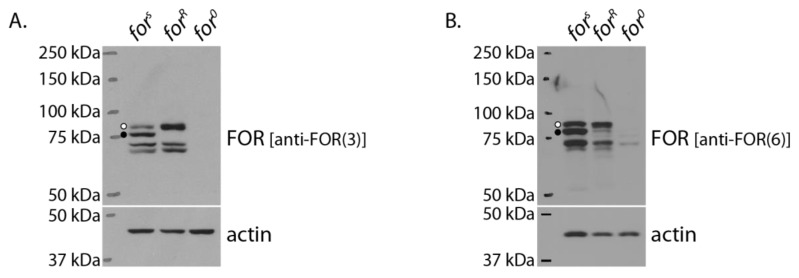
FOR expression differed between *for^s^* and *for^R^* larvae. Specifically, Western blots immunodetected with anti-FOR(3) showed a difference in the expression of the 85 and 83 kDa FOR polypeptides (open and closed circles, respectively) in *for^s^* and *for^R^* larvae (**A**). Immunodetection of whole-body lysates with anti-FOR(6) suggests the 85 and 83 kDa bands present in *for^s^* and *for^R^* larvae were P3 isoforms (**B**). *for^0^* larvae were used as negative controls for immunoprecipitation. Actin was presented as a loading control.

Finally, we characterized FOR expression in *for^R^* larvae and compared it with that of *for^s^*. Western blots of whole-body lysates showed that *for^R^* larvae differed in the expression of their 85 kDa and 83 kDa FOR bands (Figure 8A). Specifically, *for^R^* larvae consistently showed higher expression of the 85 kDa band compared with *for^s^* and lacked expression of the 83 kDa band. Immunodetection of whole-body *for^R^* lysates with anti-FOR(6) suggests that the 85 kDa band in *for^R^* was also a P3 isoform (Figure 8B).

**Figure 9:**
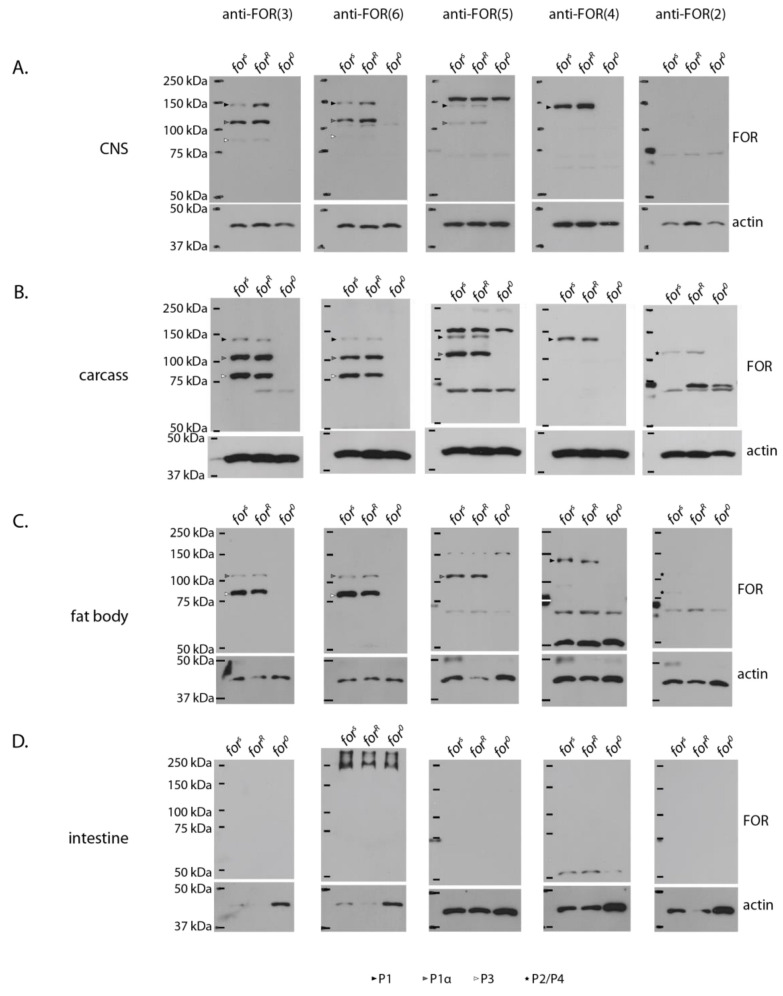
Western blots showing the FOR isoform expression in dissected organs from *for^s^*, *for^R^*, and *for^0^* larvae. *For^0^* larvae were used as negative controls for FOR expression. Organs dissected included the central nervous system (CNS, **A**), carcass (**B**), fat body (FB, **C**), and intestine (**D**). Actin was used as a loading control. Anti-FOR(3) was designed to recognize all FOR isoforms; anti-FOR(6) was designed to recognize isoforms P1, P1α, and P3; anti-FOR(5) was designed to recognize P1 and P1α; anti-FOR(4) was designed to recognize P1; while anti-FOR(2) was designed to recognize isoforms P2 and P4. FOR expression was detected in the CNS, carcass, and fat body of *for^s^* and *for^R^* larvae but not the intestine. The majority of FOR expression in the CNS, carcass, and fat body was P1, P1α, and P3. P2 and P4 isoforms were detected in the carcass and fat body.

To identify an organ responsible for the *for^s^* and *for^R^* FOR expression difference, we analyzed the *for* protein expression in the CNS, carcass, fat body, and intestine of these two genotypes. Results using anti-FOR(3) showed that the difference in the FOR expression between *for^s^* and *for^R^* larvae did not appear to be localized to either of these organs (Figure 9). Blots of dissected organs probed with isoform-specific antibodies showed that the same isoforms were expressed in the CNS, carcass, and fat body of *for^s^* and *for^R^* larvae. Specifically, the majority of FOR expression in the larval CNS, carcass, and fat body was P1, P1α, and P3. We also detected P2 and P4 specific bands with anti-FOR(2) in the larval carcass and fat body. This suggests that P2 and/or P4 were expressed in larvae and that their expression was specific to certain cell types and at levels that are not high enough to detect in whole-body extracts on a Western blot. Although we observed small differences in the protein expression of P1α and P3 between the fat body of *for^s^* and *for^R^* larvae when using anti-FOR(3), these differences were not statistically significant (*p* > 0.05, *n* = 3, one-way ANOVA, data not shown). Table 2 summarizes the spatial distribution of FOR isoforms in the larva and compares the FOR isoform expression to the published *for* gene promoter and transcript expression patterns.

## 3. Discussion

The *Drosophila* ortholog of *prkg1*, namely, the *for* gene, has long served as a model for studying the role of PKG in regulating traits such as metabolism, neurotransmission, and food intake [5,6,25,27,28]. Despite various advances in our understanding of the genetic architecture and transcriptional regulation of *for*, few studies have detailed it at the protein level [27,32,33,34]. Here, we set out to study *for*’s protein products and explore which of its predicted isoforms are expressed in vivo, and in which tissues.

Our analysis of the 22 annotated *for* transcripts from three different *for* alleles (*for^s^*, *for^R^*, and *for^BAC^::HA*) resulted in seven distinct ORFs that encoded isoforms P1, P1α, P2, P3, P4, P5, and P6, as well as an eighth ORF specific to *for^s^* transcript RM that encoded isoform P7. Despite *for* encoding at least seven putative isoforms, the majority of in vivo *for* protein expression in whole adults and larvae is derived from isoforms P1, P1α, and P3. We found that the expression of FOR isoforms differed between developmental stages and organs. Although we were not able to identify P5, P6, or P7 in our samples, the organs we analyzed were not exhaustive and our search excluded tissues suggested to have FOR expression. For example, we did not analyze the larval Malpighian tubules, which are functionally analogous to mammalian kidneys and express *for* transcripts [42,43].

In general, our in vivo results on the spatial expression of FOR isoforms align well with that of its transcripts. We detected isoforms belonging to the P1/P1α/P3 class of FOR proteins in the larval CNS, carcass, and fat body, whereas we detected isoforms belonging to the P2/P4 class of FOR proteins in the larval carcass and fat body. Similarly, published RNA-seq data on same-age third instar larvae shows exon expression for P1/P1α/P3 and P2/P4 encoding transcripts in the CNS, carcass, and fat body [39]. Interestingly, exon expression of P1/P1α/P3 and P2/P4 transcripts was also detected in the intestines of same-age third instar larvae even though we could not detect the *for* protein in that organ [39]. A gene’s transcript level does not always correlate with its protein level [44,45,46] and our inability to detect certain FOR isoforms on a Western blot in organs that show their transcript expression could have been due to low levels of translation or to low stability of those isoforms. Interestingly, the expression of *for* gene transcripts is responsive to an individual’s feeding condition [39]. As such, we speculate that the expression of certain FOR isoforms might also depend on environmental conditions that are known to influence *for*’s regulation of phenotype, such as food deprivation [25].

The higher overall expression of P1, P1α, and P3 in adults and larvae compared with all other FOR isoforms also fits with available RNA-seq data showing higher exon expressions of P1/P1α/P3 transcripts in adult males and larvae (RNA-seq by Region) [42]. It is interesting to note that adult females have higher levels of exon expression for P2/P4 transcripts compared with adult males, suggesting a possible sex-specific role for these isoforms. Indeed, exon expression data shows enrichment of P2/P4 transcripts in the ovaries of virgin and mated females (RNA-seq by Region) [42]. The role of P2 and P4 in the ovaries of *D. melanogaster* females has not yet been investigated.

Our FOR expression data in larvae also align well with cloned *for* promoter expression patterns in same-age larvae [17]. The latter data show *pr1* expression in neurons and enteroendocrine cells; *pr2* expression in glia and adult midgut precursor cells; *pr3* expression in the fat body, body wall muscle, intestinal muscle, perineural glia, enteroendocrine cells, and enterocytes; and *pr4* expression in spiracles, denticles, optic lobe neuroepithelia, hindgut epithelia, and rectal ampulla epithelia [17]. In terms of the isoforms encoded by the transcripts associated with each *for* gene promoter, *for*’s promoter expression patterns suggest the presence of P1 in the CNS and intestine; P1α and P3 in the fat body, carcass, CNS, and intestine; and P2 and P4 in the carcass, CNS, and intestine. Our Western blot results showed P1 expression in the fat body, carcass, and CNS; P1α and P3 expression in the fat body, carcass, and CNS; and P2 and P4 expression in the fat body and carcass. Although the *for* promoter expression patterns did not show fat body expression of P1, P2, or P4, we were able to detect these isoforms in this organ via Western blotting. This finding suggests the presence of additional regulatory elements outside of the cloned promoter regions that affect *for* transcription, or that *for* regulatory elements can operate independently of promoters they are closest to when regulating the expression of *for* transcripts.

It is currently not known why the expressions of P1, P1α, and P3 are more abundant than the other FOR isoforms in adults and larvae. In adult males and larvae, higher expressions of P1, P1α, and P3 proteins are correlated with higher expressions of P1/P1α/P3 transcripts (RNA-seq by Region) [42]. In addition, we speculate that P1, P1α, and P3 might ultimately be more abundant in part because of their N-terminal domain structure. Of all FOR isoforms, only P1, P1α, and P3 have N-terminal domains that possess a conserved ILz motif and Ai sequence. Studies of PKGI show that these motifs are important for regulating PKG function. The ILz motif mediates homodimerization between PKGI isoforms and provides a surface for protein–protein interactions (PPIs) between PKGI homodimers and substrates or anchor proteins [3]. The Ai sequence helps to maintain PKGI in an inactive state and influences, in trans, the binding affinities of homodimer CNBs for cGMP [3]. Experiments in vitro show that N-terminally truncated PKGI mutants demonstrate altered PKG activity. For example, N-terminally truncated PKGIα lacking its ILz motif and Ai sequence (Δ1-77) is constitutively active [8,10]. Mutant Δ1-77 can still bind two molecules of cGMP with similar overall *K_d_* compared to the wild type; however, the cooperative binding of cGMP is lost [8,9]. N-terminally truncated PKGIα lacking its ILz motif but retaining its Ai sequence (Δ1-53) is autoinhibited and is activated by cGMP in a similar manner to the wild type [12]. However, mutant Δ1-53 does not bind cGMP cooperatively and has reduced cyclic nucleotide selectivity [12]. In vivo, proteolytic cleavage of PKGIβ yields a kinase fragment, namely, PKGγ, that lacks the first 167 amino acids (Δ1-167) and is constitutively active [11]. PKGγ contains an incomplete CNB-A and a complete CNB-B and kinase domains.

We speculate that the presence of an ILz motif and Ai sequence might contribute to P1, P1α, and P3 being more tightly regulated by cGMP and that this is partly why these isoforms are more widely distributed compared with other FOR isoforms. A comparison of the P2 and P4 sequences and their expressions also supports this hypothesis. Isoforms P2 and P4 do not possess a conserved ILz motif and Ai sequence or a complete CNB-A. Despite this, P2 and P4 are speculated to show high selectivity for cGMP-dependent activation since the CNB domains immediately adjacent to the catalytic domain in PKG (CNB-B) show higher selectivity for cGMP over cAMP [47]. Isoforms P2 and P4 might, therefore, be less tightly regulated by cGMP and have higher basal activities compared with P1, P1α, and P3. In larvae, P2 and P4 are detected at low levels in specific tissues, such as the carcass and fat body. In adults, the transcript expression of P2 and P4 is high only in the ovaries of virgin and mated females (RNA-seq by Region) [42]. This lower overall expression of P2 and P4 relative to P1, P1α, and P3 may suggest a more specialized role for these isoforms. Biochemical analyses of P2 and P4 to test this have not yet been performed.

Overall, the most striking structural feature of *for*’s protein isoforms is in their differing N-terminal domains. The N-terminal domains of FOR isoforms range in amino acid length from 215 in P3 to 560 in P1. We also observed FOR isoforms with truncated P1, P1α, or P3 N-terminal amino acid sequences. Although the motifs associated with FOR N-terminal domain function have yet to be elucidated, in silico analyses using PHMMER and ProSite revealed many putative sites for PTMs (e.g., phosphorylation and glycosylation) and PPIs (e.g., coiled coils and N- and Q-rich regions). As such, the N-terminal domains of FOR’s isoforms offer many potential sources of protein regulation that could mediate the pleiotropy and plasticity of the *for* gene [25]. Interestingly, much of each isoform’s N-terminal domain is predicted to be intrinsically disordered. Intrinsically disordered regions (IDRs) can serve many regulatory roles in a protein and be involved in establishing PPIs within multienzyme complexes and switching between protein functional states [48,49]. IDRs also function in establishing biomolecular condensates through liquid–liquid phase separation, binding RNA molecules, and tethering proteins to membrane contact sites (MCSs) [50,51,52]. Thus, we speculate that some of the IDRs within the N-terminal domains of FOR may play a role in regulating isoform-specific function.

Finally, our in silico analysis of the FOR sequence identified a total of six amino acid substitutions and a single amino acid deletion in the N-terminal domains of *for^s^* and *for^R^* isoforms P1, P1α, P3, P2, and P4. Although we did not test whether the amino acid differences in *for^s^* and *for^R^* isoforms affect FOR function, it is possible that the amino acid differences could alter FOR function since the N-terminal domain of PKG has been associated with the regulation of cGMP binding affinity, substrate specificity, activation state, and subcellular localization [3,53,54,55,56,57,58,59,60]. If so, the amino acid differences in *for^s^* and *for^R^* isoforms could lead to altered cGMP signaling and, ultimately, differences in phenotype. Additionally, differences in phenotype between larvae and adults carrying the *for^s^* or *for^R^* alleles could arise from the expression of a putative FOR isoform, namely, P7, that is specific to the *for^s^* allele and is encoded by transcript RM. In our in vivo analysis of FOR, we identified a difference in expression between *for^s^* and *for^R^* larvae. Specifically, *for^R^* individuals have a higher expression of an 85 kDa P3 band compared with *for^s^*, whereas *for^s^* individuals have a higher expression of an 83 kDa P3 band compared with *for^R^*. Our results showed that this *for^s^* and *for^R^* difference in P3 expression was not associated with the CNS, carcass, fat body, or intestine. As FOR isoforms are ascribed to *for*-related traits (e.g., foraging behaviour, food intake, fat metabolism, nociception) [16,17,18,19], allelic variation in FOR amino acid sequence and isoform expression should be considered when investigating the source of *for^s^* and *for^R^* differences in phenotypes. Overall, the *for* differences we identified at the protein level contribute to and expand the pool of molecular variation that exists between individuals that carry the *for^s^* and *for^R^* alleles. Ultimately, the *for* differences we identified at the protein level could help to explain how individual differences in the aforementioned phenotypes arise in these two genotypes [25,27].

## 4. Materials and Methods

### 4.1. Fly Strains and Rearing Conditions

Fly stocks were reared on a standard cornmeal–molasses medium at 25 °C on a 12 h:12 h light/dark cycle with lights on at 800 h [61]. Adults and larvae were developmentally age-matched to reduce the potential variation in FOR expression. Adults were collected on the day of eclosion and reared in groups containing equal numbers of males and females for 3–5 days prior to experimentation. Larvae were collected within 4 h of hatching, as previously described [61], and reared to 72 ± 2 h after egg hatch (AEH) unless stated otherwise. *for^s^* and *for^R^* are the sitter and rover alleles, respectively; they are carried in strains that differ in their second chromosomes but share X and third chromosomes [16]. The *for^0^* null mutant contains a 35 kb deletion that completely removes the *for* gene and that was backcrossed into the *for^s^* genetic background [27].

### 4.2. Recombineering the for^BAC^::HA Allele

To engineer our *for^BAC^::HA* transgenic strain, we employed the galK selection/counter-selection (as in [62]) to introduce a codon-optimized HA coding sequence into the 3′ end of *for* in a bacterial artificial chromosome (BAC) containing the 35 kb locus. Generation of the 35 kb *for* locus was previously described [27]. Briefly, we PCR-amplified the GalK sequence with comTag-galK-F and comTag-galK-R primers (Table S3) and integrated it into the BAC just prior to the stop codon shared by all *for* transcripts. Codon-optimized oligos for the HA tag (YPYDVPEYA), namely, com-tagHA-F and com-tagHA-R, were synthesized with Millipore Sigma and used to replace the GalK sequence in the *for* BAC. We verified the tagged BAC using PCR, restriction digest, and Sanger sequencing. BAC integration into the attP2 landing site on the third chromosome of *Drosophila* was performed by Genetic Services Inc. using φC31 [63].

### 4.3. FOR Isoform-Specific Antibodies

Five new FOR antibodies are described here. They were generated alongside anti-FOR(3) [34], which is an antibody that recognizes a common coding region in FOR and is designed against five distinct antigenic regions in the N-terminal domains of P1, P1α, P3, P2, or P4 (Table 1). Each antigenic region was chosen, in part, because of high sequence specificity to FOR and not the two other *D. melanogaster* PKGs. The five new FOR antibodies were labelled anti-FOR(1), anti-FOR(2), anti-FOR(4), anti-FOR(5), and anti-FOR(6). Anti-FOR(1) was designed against a 37-amino-acid region in P2 and P4. Anti-FOR(2) was designed against a 77-amino-acid region in P2 and P4. Anti-FOR(4) was designed against a 147-amino-acid region in P1. Anti-FOR(5) was designed against a 63-amino-acid region in P1 and P1α. Anti-FOR(6) was designed against a 142-amino-acid region in P1, P1α, and P3. The location of each antigenic region is illustrated in Figure 4A. Methods for generating the antibodies were outlined previously [34]. Briefly, antigenic regions were cloned into pGEX-3X expression vectors (Amersham) to generate GST-FOR fusion proteins. Plasmids were transformed into BL21 (DE3) *E. coli* cells. GST-FOR was overexpressed and affinity-purified using glutathione-linked Sepharose 4B (GE Healthcare). Purified GST-FOR protein (1 mg) was used to immunize rabbits and guinea pigs. Three subsequent boosts (0.5 mg) were given at two-week intervals before terminal bleeds were collected. Antisera made in rabbit were used for anti-FOR(1), anti-FOR(4), anti-FOR(5), and anti-FOR(6). Antiserum made from guinea pig was used for anti-FOR(2). All antisera were incubated with nitrocellulose-bound GST to purify against GST-specific antibodies.

### 4.4. Dissections

Adult heads and bodies were obtained by vortexing 1.5 mL microcentrifuge tubes containing equal numbers of male and female flies that had been flash-frozen in liquid nitrogen. Sieves of different mesh sizes were used separate the heads from the bodies. Collected samples were stored at −80 °C until their homogenization in a lysis buffer. Larval organs were dissected in ice-cold 1× PBS on ice within 20 min per sample. Central nervous system (CNS) samples were dissected free of imaginal discs, carcass samples contained body wall muscles, fat body samples contained the gonads, and intestines were dissected free of Malpighian tubules. Dissected organs were placed into 1× protein loading buffer in lysis buffer and immediately frozen at −80 °C until their homogenization.

### 4.5. SDS-PAGE and Western Blotting

Samples were homogenized in a lysis buffer (50 mM Tris-HCl pH 7.5, 150 mM NaCl, 10% glycerol, 1% Triton-X 100, and 5 mM EDTA) supplemented with 1× Halt protease inhibitor (87786, Thermo Scientific, Waltham, MA, USA) and Halt phosphatase inhibitor (78420, Thermo Scientific, Waltham, MA, USA). Samples were boiled for 5 min in 2× loading buffer and 20 μg of protein per lane were loaded onto a 7.5% resolving gel and run at 100 V for 2 h for protein separation. The proteins were transferred onto a nitrocellulose membrane for 2 h at 100 V using a transfer buffer containing 10% MeOH. Western blots were performed using primary antibodies against FOR or pan-actin, and secondary antibodies were conjugated with horseradish peroxidase (HRP) diluted in a blocking solution. Bands were visualized with Amersham ECL detection reagent (GE Healthcare) and X-ray film. At least three independent biological replicates were run for each Western blot experiment and a representative blot is provided for each experiment. Lysates from *for*^0^ larvae were used as a negative control for FOR expression since homozygous *for*^0^ individuals die in the late pupal stage of development [19,27]. The molecular weight of each FOR-specific polypeptide was calculated relative to size standards on the gels. Primary antibodies used for immunodetection included anti-FOR(1) (1:10,000), anti-FOR(2) (1:3000), anti-FOR(3) (1:5000 [34]), anti-FOR(4) (1:10,000), anti-FOR(5) (1:5000), anti-FOR(6) (1:5000), and anti-actin (1:15,000; MAB1501, Millipore Sigma, St. Louis, MO, USA). Secondary antibodies included anti-guinea pig-HRP (1:15,000; code: 106-035-003, Jackson ImmunoResearch Laboratories Inc., West Grove, PA, USA), anti-rabbit-HRP (1:15,000; code: 111-035-003, Jackson ImmunoResearch Laboratories Inc., West Grove, PA, USA), and anti-mouse-HRP (1:25,000; code: 115-035-146, Jackson ImmunoResearch Laboratories Inc., West Grove, PA, USA).

### 4.6. Immunoprecipitations and Mass Spectrometry

Lysates were obtained by homogenizing 800 *for^BAC^::HA* adults or larvae over ice in a 1.6 mL lysis buffer containing 2× Halt protease inhibitor (87786, Thermo Scientific, Waltham, MA, USA) and Halt phosphatase inhibitor (78420, Thermo Scientific, Waltham, MA, USA). FOR::HA immunoprecipitations were performed by incubating supernatants with 100 μL Pierce anti-HA magnetic beads (88837, Thermo Scientific, Waltham, MA, USA) for 1.5 h at 4 °C on a rocking platform. Post-IP supernatants were kept for SDS-PAGE analysis and beads were washed twice for 10 min at 4 °C on a rocking platform with a 1 mL ice-cold lysis buffer containing 2× Halt protease and phosphatase inhibitors. Beads were washed two more times with a lysis buffer without inhibitors and boiled for 5 min in 80 μL 2× loading buffer with 0.2 M DTT to elute proteins. Homogenates of *for^s^* individuals were also IPed as a negative control. Then, 30 μL per sample were loaded in duplicate onto a 1.5 mm 7.5% resolving gel, and proteins were separated for 2 h at 100 V. Gels were stained using a 0.2% Brilliant Blue R in 50% MeOH and 10% acetic acid solution, and de-stained using a 50% methanol and 10% acetic acid solution. FOR::HA specific bands were excised, stored in 1% acetic acid at 4 °C, and sent in duplicate to the SPARC BioCentre Molecular Analysis (The Hospital for Sick Children, Toronto, ON, Canada) for sample preparation, MS, and analysis. FOR::HA bands were digested with trypsin or chymotrypsin and analyzed using liquid chromatography–tandem mass spectrometry (LC-MS/MS) on an Orbitrap Fusion Lumos mass spectrometer (Thermo Scientific, Waltham, MA, USA). MS/MS samples were analyzed using MS-Amanda Proteome Discoverer (Research Institute of Molecular Pathology, Vienna, Austria), Sequest XCorr (Thermo Fisher Scientific, Waltham, MA, USA), and X! Tandem (accessed on 10 July 2019, thegpm.org), which were set up to search the Uniprot *D. melanogaster* Database, which was modified to include the amino acid sequences of P1::HA and P3::HA. Scaffold (Proteome Software Inc., Portland, OR, USA) was used to validate the MS/MS-based peptide and protein identifications. Peptide identifications were accepted if they could be established at greater than 95% probability. Peptide probabilities were assigned by the Peptide Prophet algorithm [64] with Scaffold delta-mass correction. Protein identifications were accepted if they could be established at greater than 95% probability and contained at least 2 identified peptides. Protein probabilities were assigned by the Protein Prophet algorithm [65]. Proteins that contained similar peptides and could not be differentiated based on MS/MS analysis alone were grouped to satisfy the principles of parsimony. To estimate the likelihood that a given amino acid sequence was present in a FOR::HA band, we calculated the amino acid relative abundances within each sample by dividing the number of times an amino acid sequence was detected within a sample by the highest number of times that any sequence was detected for that same sample. Relative abundances are presented as values that range from 0.0 to 1.0.

## 5. Conclusions

Overall, our in vivo results show that various protein isoforms are expressed by the *for* gene and that their expression differs between organs, developmental stages, and genotypes. We found that the majority of in vivo FOR expression arose from isoforms P1, P1α, and P3 despite *for* coding for eight distinct protein isoforms. Interestingly, unlike the other FOR isoforms, the P1, P1α, and P3 isoforms possessed an ILz motif and Ai sequence in their N-terminal domains that are conserved in mammalian PKGs. Although our study did not characterize the biochemical properties of the different FOR isoforms, future studies of FOR could focus on identifying differences in isoform function and characterizing isoform-specific regulation of phenotypes influenced by the *for* gene. Our results also showed that FOR expression differed between larvae carrying the *for^s^* (sitter) and *for^R^* (rover) alleles. Sitter and rover are two allelic variants of *for* that differ in many food-related traits. While our study did not determine a functional role for the difference in FOR expression between sitter and rover allelic variants, future studies of *for^s^* and *for^R^* should consider it when investigating *for*’s role in the regulation of individual differences in phenotypes.

## Figures and Tables

**Table 1:** List of antigenic regions used to generate isoform-specific FOR antibodies.

Antibody	Antigen Sequence	Isoform Specificity
Anti-FOR(1)	SPPKIKENLSKSSSAYSTFSSAAEDSQDQVVICQQPQ (P2 residues 191 to 227)	P2, P4
Anti-FOR(2)	RRLSLEQAIEGLKLEGEKAVRQKSPQISPAASSNGSSKDLNGEGFCIPRPRLIVPVHTYARRRRTGNLKEQSSGGQE (P2 residues 286 to 362)	P2, P4
Anti-FOR(3) [34]	WFDGFYWWGLQNCTLEPPIKPAVKSVVDTTNFDDYPPDPEGPPP (catalytic domain)	P1, P1α, P2, P3, P4, P5, P6, P7
Anti-FOR(4)	HSSTTVDAPPRPADVDVATVPVATTAPPPQQPVSNLFYADYQKLQPAIIDRDWERDRDTDTDTRSEAKPPDIVEHIEPVEEQRQIHTQIQSPAEIQIQIPPTPPAPSIQIQIQQRYRRHSSAEDRNLNTRRNDSNITEALRKAASMQ (P1 residues 25 to 171)	P1
Anti-FOR(5)	QQELQLQQRYQQLQQLQAQTQGLYTSQGSPVLYHQPSPGSSQPVAIPGATCHSPTQLQPPNTL (P1 residues 278 to 340)	P1, P1α
Anti-FOR(6)	ISGCTPSGTGGSATPSPVGLVDPNFIVSNYVAASPQEECFIQIIQAKELKIQEMQRALQFKDNEIAELKSHLDKFQSVFPFSRGSAAGCAGTGGASGSGAGGSGGSGPGTATGATRKSGQNFQRQRALGISAEPQSESSLLL (P1 residues 351 to 492)	P1, P1α, P3

**Table 2:** Summary of the *for* gene transcript and protein expression.

	CNS	Carcass	Fat Body	Intestine
FOR isoform expression by *for* promoter Gal4 expression [17]	P1, P5, P7 * (*pr1*) P1, P5 (*pr2*) P1α, P3 (*pr3*) P2, P4, P6 (*pr4*)	P1α, P3 (*pr3*) P2, P4, P6 (*pr4*)	P1α, P3 (*pr3*)	P1, P5, P7 * (*pr1*) P1, P5 (*pr2*) P1α, P3 (*pr3*) P2, P4, P6 (*pr4*)
FOR isoform expression by modENCODE RNA-seq by region (FlyBase)	P1/P1α/P3 (exon 4) P2/P4 (exon 7)	P1/P1α/P3 (exon 4) P2/P4 (exon 7)	P1/P1α/P3 (exon 4) P2/P4 (exon 7)	P1/P1α/P3 (exon 4) P2/P4 (exon 7)
FOR isoform expression	P1, P1α, P3	P1, P1α, P2, P3, P4	P1α, P2, P3, P4	No detectable expression

* Isoform P7 is specific to the *for^s^* allele.

## Data Availability

Data is contained within the article or Supplementary Material.

## Supplementary Materials

The following supporting information can be downloaded from https://www.mdpi.com/article/10.3390/ijms241210219/s1.
