# Supplementary material for: Characterizing the Protein Isoforms of foraging (for), the PKGI Ortholog in Drosophila melanogaster
Source: Int J Mol Sci. 2023 Jun 16;24(12):10219. doi: 10.3390/ijms241210219 (PMC10299703; doi:10.3390/ijms241210219)
Supplement: Supplementary file 1 [file ijms-24-10219-s001.zip › ijms-2393093-supplementary.pdf]

Characterizing the Protein Isoforms of *foraging* (*for*), the PKGI Ortholog in *Drosophila melanogaster*

Oscar E. Vasquez [1,*], Aaron M. Allen [2], Anthony K.-C. So [3], Quynh H. Nguyen [4], Henry M. Krause [5], Joel D. Levine [3,6] and Marla B. Sokolowski [1,6,*]

1. Department of Ecology and Evolutionary Biology, University of Toronto, Toronto, ON M5S 3B2, Canada
2. Centre for Neural Circuits and Behaviour, Oxford University, Oxford OX1 3SR, UK; aaron.allen@dpag.ox.ac.uk
3. Department of Biology, University of Toronto at Mississauga, Mississauga, ON L5L 1C6, Canada; anthony.kc.so@gmail.com (A.K.-C.S.); joel.levine@utoronto.ca (J.D.L.)
4. Department of Biochemistry, University of Toronto, Toronto, ON M5S 1A8, Canada; qhuong.nguyen@mail.utoronto.ca
5. Department of Molecular Genetics, University of Toronto, Toronto, ON M5S 3E1, Canada; h.krause@utoronto.ca
6. Child and Brain Development Program, Canadian Institute for Advanced Research (CIFAR), Toronto, ON M5G 1M1, Canada

* Correspondence: oscar.vasquez@utoronto.ca (O.E.V.); marla.sokolowski@utoronto.ca (M.B.S.)

Supplemental Figures and Tables: 4

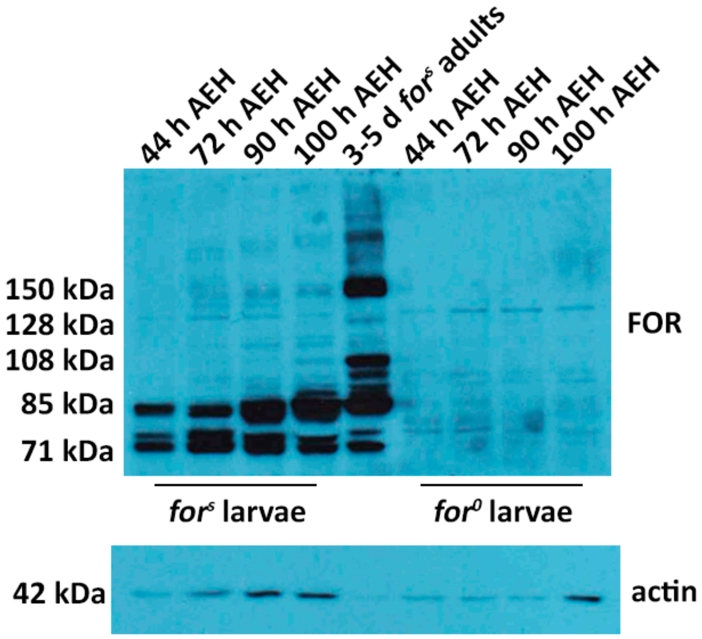

Figure S1. FOR expression changes through development. Western blot of FOR expression from whole-body lysates of *D. melanogaster* larvae at different developmental ages after egg hatch (AEH) and adults. FOR bands detected in second instar larvae aged 44 h AEH do not differ from those detected in mid-third instar larvae aged 72 h AEH. FOR expression changes by 90 h AEH and continues to change as larvae enter the wandering stage at 100 h AEH. FOR

expression is stable in the adult stage but is shown only for adults aged 3-5 days (d) old. Blot was probed with anti-FOR(3). Actin is presented as a loading control.

Table S1. Prosite analysis of FOR isoform P1 amino acid sequence.

Predicted post-translational modification	Enzyme	Amino acid residues
Phosphorylation (T12)	PKC	TKR (12-14)
Phosphorylation (T153)	PKC	TRR (153-155)
Phosphorylation (S348)	PKC	SLR (348-350)
Phosphorylation (T465)	PKC	TRK (465-467)
Phosphorylation (T685)*	PKC	TIK (685-687)
Phosphorylation (S799)*	PKC	SSR (799-801)
Phosphorylation (S824)*	PKC	SEK (824-826)
Phosphorylation (T843)*	PKC	TFK (843-845)
Phosphorylation (T876)*	PKC	TTR (876-878)
Phosphorylation (T927)*	PKC	TGR (927-929)
Phosphorylation (T28)	CK2	TTVD (28-31)
Phosphorylation (T83)	CK2	TDTD (83-86)
Phosphorylation (T87)	CK2	TRSE (87-90)
Phosphorylation (S115)	CK2	SPAE (115-118)
Phosphorylation (S144)	CK2	SSAE (144-147)
Phosphorylation (S145)	CK2	SAED (145-148)
Phosphorylation (T247)	CK2	THSD (247-250)
Phosphorylation (S384)	CK2	SPQE (384-387)
Phosphorylation (S420)	CK2	SHLD (420-423)
Phosphorylation (T691)*	CK2	TQEE (691-694)
Phosphorylation (S744)*	CK2	SNLD (744-747)
Phosphorylation (T795)*	CK2	TNGD (795-798)
Phosphorylation (S824)*	CK2	SEKE (824-827)
Phosphorylation (T843)*	CK2	TFKD (843-846)
Phosphorylation (T975)*	CK2	TGSD (975-978)
Phosphorylation (S1058)*	CK2	SVVD (1058-1061)
Phosphorylation (T1063)*	CK2	TNFD (1063-1066)
Phosphorylation (T1081)*	CK2	TGWD (1081-1084)
Phosphorylation (S144)	PKA/PKG	RRHS (141-144)
N-linked glycosylation (N156)	n/a	NDSN (156-159)
N-linked glycosylation (N159)	n/a	NITE (159-162)
N-linked glycosylation (N193)	n/a	NNTN (193-196)
N-linked glycosylation (N208)	n/a	NNSN (208-211)
N-linked glycosylation (N772)*	n/a	NLTD (772-775)
N-linked glycosylation (N998)*	n/a	NITR (998-1001)
N-linked glycosylation (N1002)*	n/a	NASN (1002-1005)
N-linked glycosylation (N1045)*	n/a	NCTL (1045-1048)

Phosphorylation (Y536)*	Tyrosine kinase	REIVDCMY (529-536)
Phosphorylation (Y849)*	Tyrosine kinase	KTFKDKKY (842-849)

* PTMs found in amino acid sequence shared between isoforms.

Table S2. Prosite analysis of FOR isoform P4 amino acid sequence.

Predicted post-translational modification	Enzyme	Amino acid residues
Phosphorylation (T92)	PKC	TIK (92-94)
Phosphorylation (T155)	PKC	TER (155-157)
Phosphorylation (S168)	PKC	SVK (168-170)
Phosphorylation (S173)	PKC	SLR (173-175)
Phosphorylation (T188)	PKC	TLR (188-190)
Phosphorylation (T261)	PKC	TVR (261-263)
Phosphorylation (S284)	PKC	SQR (284-286)
Phosphorylation (S321)	PKC	SSK (321-323)
Phosphorylation (T532)*	PKC	TIK (532-534)
Phosphorylation (S646)*	PKC	SSR (646-648)
Phosphorylation (S671)*	PKC	SEK (671-673)
Phosphorylation (T690)*	PKC	TFK (690-692)
Phosphorylation (T723)*	PKC	TTR (723-725)
Phosphorylation (T774)*	PKC	TGR (774-776)
Phosphorylation (T92)	CK2	TIKE (92-95)
Phosphorylation (S140)	CK2	SIQE (140-143)
Phosphorylation (S211)	CK2	SAAE (211-214)
Phosphorylation (S321)	CK2	SSKD (321-324)
Phosphorylation (T538)*	CK2	TQEE (538-541)
Phosphorylation (S591)*	CK2	SNLD (591-594)
Phosphorylation (T642)*	CK2	TNGD (642-645)
Phosphorylation (S671)*	CK2	SEKE (671-674)
Phosphorylation (T690)*	CK2	TFKD (690-693)
Phosphorylation (T822)*	CK2	TGSD (822-825)
Phosphorylation (S905)*	CK2	SVVD (905-908)
Phosphorylation (T910)*	CK2	TNFD (910-913)
Phosphorylation (T928)*	CK2	TGWD (928-931)
N-linked glycosylation (N198)	n/a	NLSK (198-201)
N-linked glycosylation (N266)	n/a	NQTT (266-269)
N-linked glycosylation (N282)	n/a	NASQ (282-285)
N-linked glycosylation (N319)	n/a	NGSS (319-322)
N-linked glycosylation (N619)*	n/a	NLTD (619-622)
N-linked glycosylation (N845)*	n/a	NITR (845-848)
N-linked glycosylation (N849)*	n/a	NASN (849-852)
N-linked glycosylation (N892)*	n/a	NCTL (892-895)
Phosphorylation (S246)	PKA/PKG	KRVS (243-246)
Phosphorylation (S289)	PKA/PKG	RRLS (286-289)

Phosphorylation (T350)	PKA/PKG	RRRT (247-350)
Phosphorylation (Y696)*	Tyrosine kinase	KTFKDKKY (689-696)

* PTMs found in amino acid sequence shared between isoforms.

Table S3. Oligos used for recombineering of *for^{BAC}::HA* allele.

Oligo name	Sequence
comTag-galK-F	ATCCTGAGGGTCCGCCGCCAGATGATGTCACTGGATGGGACAAGGACTTC
comTag-galK-R	CAGAAGCGTTTAGAGAGCATCGTCTAGGAAACGGGTTCTGATTCTCCTCA
com-tagHA-F	GTCCGCCGCCAGATGATGTCACTGGATGGGACAAGGACTTCTACCCCTACGACGT GCCCGAGTACGCCTGAGGAGAATCAGAACCCGTTTCCTAGACGATGCTCTCTAA
com-tagHA-R	TTAGAGAGCATCGTCTAGGAAACGGGTTCTGATTCTCCTCAGGCGTACTCGGGCAC GTCGTAGGGGTAGAAGTCCTTGTCCCATCCAGTGACATCATCTGGCGGCGGAC
